# A Platform for Processing Expression of Short Time Series (PESTS)

**DOI:** 10.1186/1471-2105-12-13

**Published:** 2011-01-11

**Authors:** Anshu Sinha, Marianthi Markatou

**Affiliations:** 1 Department of Biomedical Informatics, Columbia University, New York, NY, USA; 2 Department of Biostatistics, Columbia University, New York, NY, USA

## Abstract

**Background:**

Time course microarray profiles examine the expression of genes over a time domain. They are necessary in order to determine the complete set of genes that are dynamically expressed under given conditions, and to determine the interaction between these genes. Because of cost and resource issues, most time series datasets contain less than 9 points and there are few tools available geared towards the analysis of this type of data.

**Results:**

To this end, we introduce a platform for Processing Expression of Short Time Series (PESTS). It was designed with a focus on usability and interpretability of analyses for the researcher. As such, it implements several standard techniques for comparability as well as visualization functions. However, it is designed specifically for the unique methods we have developed for significance analysis, multiple test correction and clustering of short time series data. The central tenet of these methods is the use of biologically relevant features for analysis. Features summarize short gene expression profiles, inherently incorporate dependence across time, and allow for both full description of the examined curve and missing data points.

**Conclusions:**

PESTS is fully generalizable to other types of time series analyses. PESTS implements novel methods as well as several standard techniques for comparability and visualization functions. These features and functionality make PESTS a valuable resource for a researcher's toolkit. PESTS is available to download for free to academic and non-profit users at http://www.mailman.columbia.edu/academic-departments/biostatistics/research-service/software-development.

## Background

A frequent goal of high-throughput biological studies, in general, and microarray studies, in particular, is the identification of genes that show differential expression between phenotypes (e.g. cancer vs. no cancer). Microarray experiments are used in a wide variety of studies to understand the mechanisms governing variation in complex traits [1], for example, in studies of treatment effects on diseases [2]. Using microarray technology, mRNA expression data can be gathered on whole genomes or tens of thousands of unique DNA sequences at a time. And this data provides a snapshot of gene activity in a particular sample at a particular time. This snapshot, or cross-sectional point of view, has dominated microarray research [3] and much has been published on the identification of differentially expressed genes. Taking a snapshot of the expression profile following a new condition can reveal some of the genes that are specifically expressed under the new condition. However, in order to determine the complete set of genes that are expressed under these conditions, and to determine the interaction between these genes, it is necessary to measure a time course of expression experiments [4]. Time-dependent, or temporal, microarray profiles look at the expression of genes over a time domain, with the goal of taking a closer view at gene expression profiles to understand their characteristics. They provide an additional layer of information and an important characterization of gene function, as biological systems are predominantly developmental and dynamic.

Typical characteristics of microarray time course data are: 1) sparsity, in terms of both the number of replicates per sample and the number of time points per replicate and 2) irregularly spaced time points. Although there have been temporal microarray studies with as many as 80 time points, almost all are much shorter. In fact, Ernst et al. (2005) [5] found that more than 80% of all time series datasets they surveyed contained less than 9 points. The primary reason why short time-series datasets are so common is expense - a limiting factor for most researchers. Additionally, it can be difficult to obtain large quantities of biological material. These factors can similarly limit the number of replicates tested and drive the use of irregularly spaced time points as well.

The purpose of this paper is to introduce the Processing Expression of Short Time Series (PESTS) platform, designed for the complete analysis of short time series gene expression datasets. PESTS provides a set of methods targeted to the analysis of sparse and irregularly-spaced time course microarray expression data making minimal assumptions about the underlying process that generated the data. It is designed specifically for the unique methods we have developed for significance analysis, multiple test correction and clustering of short time series data. Although PESTS was specifically designed for short microarray time series analyses, it is generalizable to other, longer time series analyses. Together with its implementation of several standard techniques and its visualization capabilities, users may find PESTS to be a useful tool for time series data analysis with or without PESTS-specific algorithms.

Much of the work on significance analysis of time series expression experiments uses methods originally developed for static or uncorrelated data [6-8]. While biologically relevant results may be found, these methods ignore the trend or sequential nature of time courses. At the same time, static methods do not allow us to leverage the attributes of time course data. More recently, several algorithms have been developed [3,9,10] which use model-based techniques to determine significant genes, accounting for time-dependence, but are generally more appropriate with longer time series. Non-parametric approaches have also been devised including those in [11,12]. Similarly, clustering techniques for time course data have gone through a similar evolution from static techniques [6,13] to a host of model-based techniques [4,14,15], to non-parametric methods targeting short time series [5,16] with analogous advantages and pitfalls.

The fundamental principle behind the time series methods developed for PESTS is to appropriately use expression profiles and dependence across time points to determine salient genes and gain biological insight about them while accounting for sparsity in the data. Instead of using model-based techniques which do account for time dependence but generally tend to be inappropriate in cases of sparsity, PESTS methods summarize profiles using an innovative set of features. Features summarize short gene expression profiles, inherently incorporate dependence across time, and allow for both full description of the examined curve and missing data points. They are based on the structural characteristics of the time course data and reflect a clear link with subject-matter considerations, capturing the "global picture" of an admittedly short time horizon of expression. In the case of short time series, features are used as a dimension augmentation technique. By contrast, this algorithm could also be extendable to longer time series through the use of features which provide dimension reduction such as autocorrelation functions, skewness, kurtosis, etc. as well as the descriptive features presented here. These biologically relevant features or curve summarization measures are then used for significance analysis or clustering. We provide brief summaries for these methods in the context of the interface description next and further information can be found in [17,18].

In this paper, we will discuss details of the PESTS platform as well as give brief overviews of the relevant methodologies used and evaluation. First, we give implementation details and briefly discuss data requirements for using the platform. Then we give an overview of the interface, as well as the implemented visualization tools. Lastly, we compare the platform to other available resources for both significance analysis of time course data and clustering.

### Implementation

The focus of this work is on time series data that is both sparse and irregularly spaced. Thus, the methods presented are implicitly tailored to these data characteristics. Here, we note our other guiding principles. First, the interface is designed for both paired and unpaired data. For significance analysis, the data must have more than one treatment, allowing for comparison. While paired data has the same number of replicates per treatment by definition, unpaired data is not required to. Furthermore, any given replicate can have measurements taken at different time points. In other words, for a given analysis, there are *i *= 1,..., *I *treatments and *r*_*i *_= 1, ..., *R*_*i *_replicates for each treatment. Additionally, there are $t_{r_{i}}=1,\;...\;,T_{r_{i}}$ time points for each replicate in each treatment. In the case of paired data, for a given treatment *i *≠ *j*, *R*_*i *_= *R*_*j *_but this may or may not be the case for unpaired data. In either case, time points of measurement may not be the same, so for a treatment *i *and replicate $r\neq s,either\;\;T_{r_{i}}=T_{s_{i}}or\;T_{r_{i}}\neq T_{s_{i}}$.

PESTS is implemented entirely in Java http://www.java.com and will work with any operating system supporting Java 6 or later. Advantages to using Java for this platform are that it is flexible, freely distributed, provides comprehensive graphical interface capabilities, implementations are platform independent, and the use of an interface does not require expertise in any programming language, statistical or otherwise, for the user. Further, Java is well-suited to memory management tasks, critical in data-intensive analyses such as microarray analyses. Because of the large open-source community, many implementations of methods found in standard statistical packages were available to us for development. However, we do note some limitations in this area, so some methods were implemented from scratch - most notably, the clustering algorithms. Several third party libraries were used to support the application. The Java Statistical Classes (JSC) http://www.jsc.nildram.co.uk/ package was used for some of the standard statistical computations. Foxtrot http://foxtrot.sourceforge.net/ was used for thread management. JFreeChart http://www.jfree.org/jfreechart/ provided implementations for plot rendering. The JExcel API http://jexcelapi.sourceforge.net/ was used to generate excel spreadsheets for saving results. Lastly, EaSynth http://www.easynth.com/ was used for the look and feel of the application.

**Figure 1:**
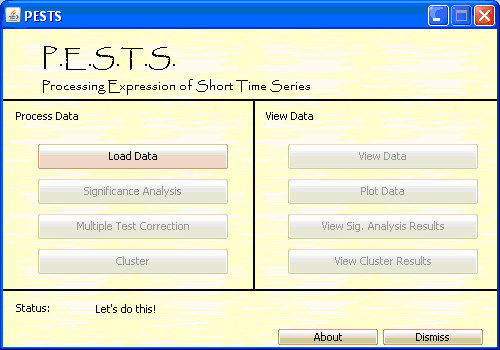
**Main PESTS screen**.

The main PESTS interface is structured as a portal of available functionality for processing and viewing data. This screen is shown in Figure 1. At launch, the only available option is to load files. And options become enabled as the user performs the prerequisite operations. We note here that PESTS assumes the data entered is logged (base 2), but allows the user to indicate that the software should log the data when loading it. This is important because the data is handled as such. Also, we note here that each screen in the interface provides a help button to guide the user.

**Figure 2:**
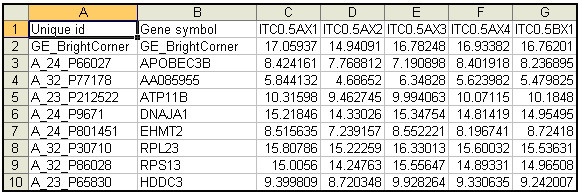
**Example of a portion of a gene expression data input file for PESTS**.

PESTS requires as input two files. The first file is a tab-delimited file of gene expression data. The second file is a tab-delimited label file of the associated metadata for the arrays. The expression data file includes unique probe identifiers, optional gene symbols, and data values. An example is given in Figure 2 below. The first line contains the header for the probe identification (ID) column (any name), optionally the header for the symbol column (any name) and the names of the arrays delimited by tabs. The remaining lines contain the probe IDs, optional gene symbols, and then data delimited by tabs. We note here that the software can be used with any type of data (one-color, two-color, or any other type of data) as long as it is formatted for the software. Moreover, many standard software are available for performing data transformations such as normalization and these choices should be made prior to data input. The only assumption made by the software is that the data is logged (base 2).

**Figure 3:**
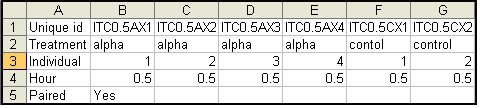
**Example of a portion of a label input file for PESTS**.

The label file contains the covariate information which indicates the treatment, replicate number and time point of each array as well as a specifier indicating whether the data is paired. An example is given in Figure 3 below. The first line contains the header for the probe id column (any name), optionally the header for the symbol column (any name) and the names of the arrays delimited by tabs. The arrays do not need to be in the same order as the expression file. The second row indicates the treatment for each array. For example, Figure 2 shows two treatments, alpha and control, representing two different treatments of the data. The third row indicates the replicate number (an integer, but does not need to be ordered) for each array. If measurements are paired, the replicate numbers should match across treatments. The fourth row indicates the time point of the array. Time points are not required to match across replicates but should obviously overlap in range to facilitate analysis. The optional fifth row indicates if the data is paired by 'yes' or 'no'. If the row is not there, the application defaults to unpaired.

**Figure 4:**
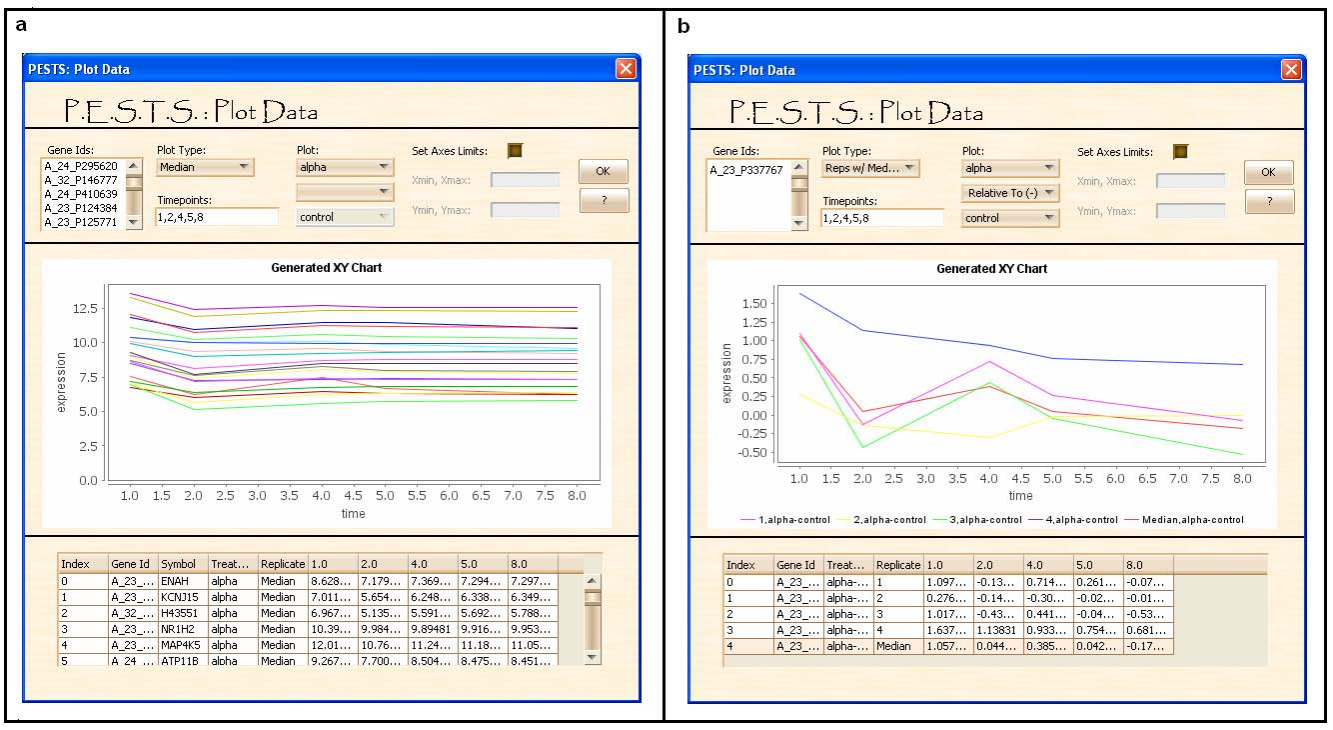
**Plotting data in PESTS**. (a) shows median alpha expressions for a set of given genes. (b) shows the replicate and median expression for a given gene for alpha relative to control treatment.

**Figure 5:**
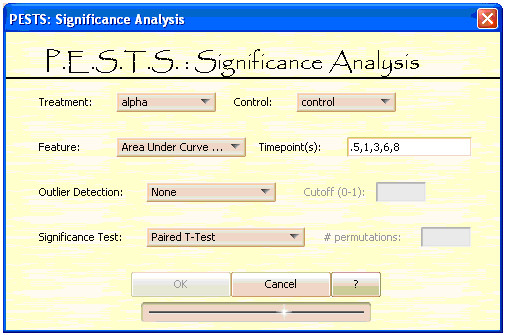
**PESTS Significance Analysis Screen**.

**Figure 6:**
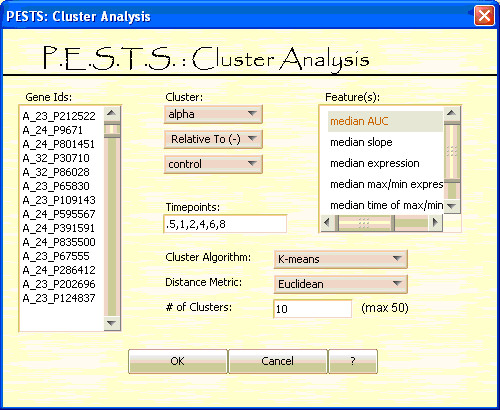
**Cluster Analysis Screen**.

Once the files are loaded, users are then able to view the raw expression data, plot the data as in Figure 4(a) and 4(b), perform significance analysis (Figure 5), or perform cluster analysis (Figure 6). While clustering is usually done based on results of significance analysis, it can be done directly as well if the user has a subset of IDs to examine. As shown in Figure 4(a) and 4(b), the user inputs parameters for plotting in the top panel of the interface. The interface allows options for plotting all the replicates for one gene or plotting median (or mean) expressions for a set of genes. The user must input the time points desired for plotting which do not have to be the same as the time points of measurement. If some of the time points desired are within the observation horizon but measurements have not been taken, PESTS can linearly interpolate the appropriate measurement. We note that the interpolation must be within the range of the observation. Further, we discourage interpolating too many time points because this assumes a linear relationship in the data which may or may not be true. As a rough guideline, no more than 10% of the total number of time points should be interpolated. When plotting median data, the median over all replicates is taken. Finally, the user can select the desired treatment to plot or plot two treatments together. If the data are paired, the user can plot one treatment relative to another. The plotting interface shows the generated plot in the middle panel of the interface. And the bottom panel shows the data used to generate the plot, including the id, symbol, treatment, replicate and the expression at each of the selected time points. This plotting interface is designed to be flexible and intuitive. Furthermore, it allows for quick examination of results found through the analysis methods.

Figure 5 shows the interface for the significance analysis method. The user first selects the treatments to compare and then chooses a feature, or data summarization measure, of the gene expression profile to use for comparison. The replicates as denoted in the covariate file are then used for comparison. Obviously, as in standard statistical comparisons, the more replicates there are, the more power in the analysis. However, we suggest not using less than 3 replications for comparison. On the other hand, we note here that the clustering piece of the software can be executed with or without replications. The available choices are the signed Area Under the Curve (AUC), the slope between 2 time points, and a particular time point. The signed AUC is a good choice when the biological question has to do with the overall change in expression over a chosen time frame. The slope over a time period can be used to compare the rate of change, and the time point is a good choice when a particular time is known to show maximal change. The time point field allows the user to input the time points to use to calculate the feature, thereby specifying a period of interest. Time points should be entered as discussed previously. The null hypothesis of no differential expression is tested using standard statistical tests. Both parametric and non-parametric tests are listed, as well as tests for paired data if applicable. Parametric tests included are the t-test and the paired t-test which can be used when the distribution of the selected feature is approximately normal or assumed to be approximately normal. The non-parametric tests include the Wilcoxon test, the Mann-Whitney U test, the permutation t-test and the permutation paired t-test which do not make assumptions about the distribution of the feature and are thus less powerful. All of these tests assume independent samples. The user can select the test from the drop down box. The user can also select options for outlier removal. We provide three methods for outlier removal. The first two [19] approximate the variance of the selected feature and use the difference between the mean and median to find outliers. The last, Dixon's Extreme Value Test [20], is specific to cases where sample size<25 and can be used to find outliers in both tails.

**Figure 7:**
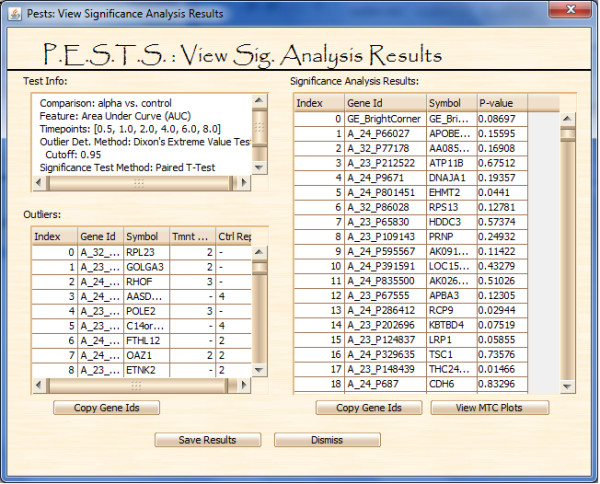
**PESTS screen for viewing significance analysis results**.

After this, the main portal activates the multiple test correction button and the view significance analysis results button. Figure 7 shows the results screen. The top left panel summarizes the test information. The bottom left panel shows the removed outlier genes and the right panel shows the significance results. Gene ids can be easily selected and then plotted to examine results. At this point, no multiple test correction has been performed but the user can view generic multiple test information using the 'View MTC Plots' button. Additionally, significance results can be saved using the 'Save Results' button.

**Figure 8:**
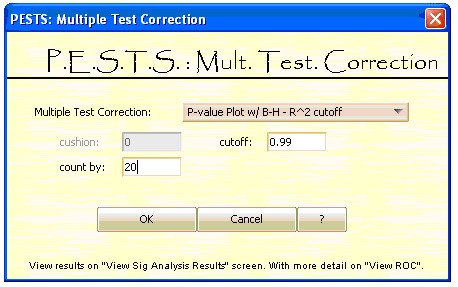
**Multiple Test Correction Screen**.

The multiple test correction screen is shown in Figure 8. The user first selects the multiple test correction method and then inputs required for the specific test. The PESTS platform provides standard corrections such as the Bonferroni correction [21] and the Benjamini-Hochberg correction [22]. The other tests listed provide novel methods [18] for estimating the number of truly null hypotheses being tested in the data. Briefly, an important step in measuring significance in the case of a large number of tests is estimation of the number of true null hypotheses. The practical issues of how well we can identify m-m_0 _differentially expressed genes (where *m *= the total number of genes and *m_0 _*= the number of genes for which the null hypothesis is true) is addressed here and used to adjust the measure of the false discovery rate (FDR) for each gene. We present two methods for *m_0 _*estimation here. The first uses a p-value plot [23], defined as *N_p _vs. 1-p*, where *p *is the p-value (from 0 to 1) and *N_p _*is defined as the number of p-values (across the entire dataset) that are greater than *p*. The p-value plot was first suggested by Schweder and Spjotvoll (1982) [23] to determine a cutoff point for differentiating significant hypotheses from non-signficant hypotheses. Because this plot should be approximately linear when all hypotheses are truly null, the points that deviate from linearity correspond to null hypotheses that should be rejected. We devised an algorithm based on the increase of the R^2 ^coefficient which describes how well a straight line is fitted. More details can be found in [18]. And this is used to update the Benjamini-Hochberg estimated false discovery rate for each gene. Second, we use a CDF plot which should also be linear when all hypotheses are truly null. Here we use a ratio of the expected area under the curve to the actual area to estimate the proportion of null hypotheses. More information on the calculations can be found at [18].

**Figure 9:**
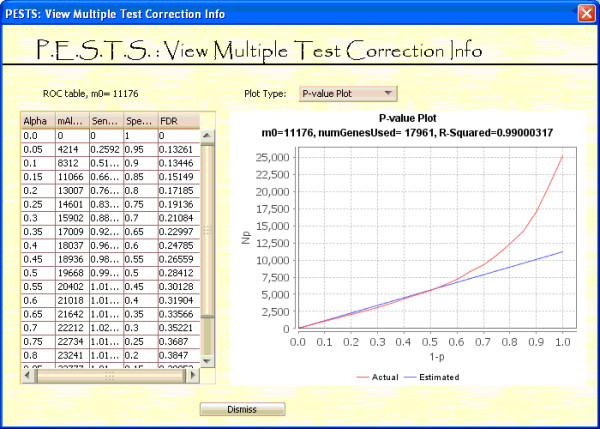
**View Multiple Test Correction Screen**.

Figure 8 shows the screen to view the multiple test correction. The left panel indicates the calculated *m_0 _*and the corresponding estimates for sensitivity, specificity and false discovery rate for various levels of significance. These can be used to determine an appropriate threshold of significance for a particular dataset and an estimated *m_0_*. The right panel is a graphing panel that can show the ROC plot, the p-value plot or the CDF plot.

Finally, the user can perform cluster analysis. The clustering screen is shown in Figure 6. The left panel is used to input the gene probe ids to be clustered. The user also needs to select the treatment(s); if the data are paired, the user can cluster the difference between two treatments. The top right panel lets the user select the feature(s) to be used for clustering. As with the significance analysis, the data are clustered using features of the gene expression curve in order to account for sparsity and incorporate dependence inherent in time course data. The current list of features is: the signed AUC, the slope, the raw expression, the maximum and minimum expressions, the time of the maximum and minimum expressions, and the steepest positive and negative slopes. Features are summarized using either the mean or median across replicates. In the sparse-data context, we use feature selection as a dimension augmentation technique to effectively and appropriately describe the curve and provide the most complete description of a time series as possible. The clustering features we use here are based on the structural characteristics of the time course data and meant to reflect a clear link with subject-matter considerations and the questions under study. The user should select the feature(s) that are germane to their particular analysis. Again, the user identifies the time points to use for calculating the features. Lastly, the user selects the clustering algorithm (K-means or PAM), the distance metric (Euclidean or Manhattan) and the number of clusters. The question of the appropriate number of clusters can be addressed manually with our system. We suggest running the algorithm over a reasonable set of *k*s and choosing the optimal *k *as the clustering with the highest average silhouette [24].

**Figure 10:**
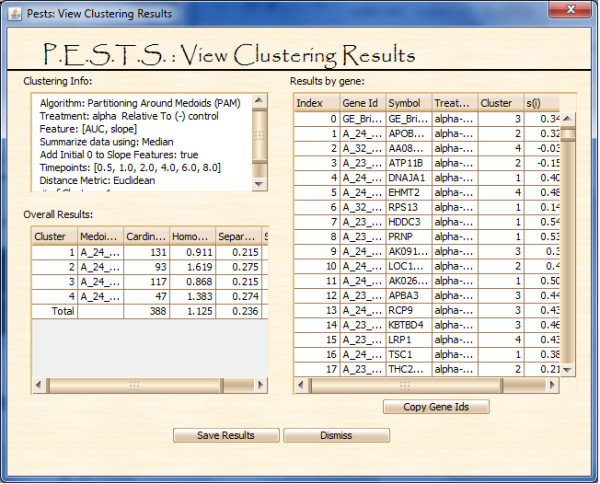
**View Clustering Results Screen**.

Clustering results can be viewed as shown in Figure 10. The top left panel shows the clustering parameters used. The bottom left panel gives overall cluster information and results for computational evaluation metrics. For cluster tightness, we show homogeneity. And to measure overall cluster structure and separation, we display separation and silhouette. All methods are described in [18]. Double-clicking on the cluster line item will pop up a plot of the cluster. Finally, the right panel gives the cluster assignments for each gene as well as their individual silhouettes and nearest neighboring cluster. Gene IDs can also be copied into the plotting screen to view the overall cluster profile. Additionally, clustering results can be saved using the 'Save Results' button.

## Results and Discussion

There are few software platforms available for the purposes of short time-series data analysis. In terms of both significance analysis and clustering, PESTS is the only platform we are aware of that does both.

For identifying differentially expressed genes, the available options are Significance Analysis of Microarrays (SAM) [11], Extraction of Differential Gene Expression (EDGE) [25], and maSigPro [26] which is incorporated in to the Serial Expression Analysis (SEA) [27] platform, a web-based tool for analysis. EDGE is an R-based platform which models time course data using splines and then uses model fit information to determine significance. It also uses a method for *m_0 _*estimation to improve FDR calculations. Given that this method requires model-fitting, it may be more suitable to longer time series or data sets with many replicates, which allow for accurate estimation of model parameters. Similarly, maSigPro is a two-regression step approach targeted to determining differences in time course expression over multiple treatments of the data. The reliance on model fitting with a specific functional form for the time element and a two-step regression strategy suggests limitations, similar to those met in other model-based approaches, when applied to short time series. Additionally, maSigPro does not perform *m_0 _*estimation. SAM is an R-based excel plugin tool. It is similar to PESTS in that its time series method uses features such as the signed AUC or slope across time points, and it uses the SAM test for significance. SAM also performs *m_0 _*estimation for multiple test correction. However, using PESTS, other standard tests of significance can be applied using information about the data distribution. Furthermore, the PESTS interface allows more flexibility and usability in time point selection. A user would need to modify the input files in order to look at different periods of time with any of these platforms. Both EDGE and SAM use asymptotic *m_0 _*estimation methods which are useful but may not be optimal in certain datasets. Additionally, PESTS provides information about the sensitivity and specificity to aid the user in selecting a reasonable threshold for significance. It also provides methods for outlier detection and removal. Genes with outliers are removed from testing, increasing the reliability of results.

For clustering, there are several more options. Order Restricted Inference for Ordered Gene Expression data (ORIOGEN) [28] uses user-defined candidate temporal profiles based on mean expression measurements at each time point and then assigns genes to the best-fitting pre-defined profile. This approach uses bootstrapping to asses significance for each gene, and thus requires more than a handful of (independent) replicates. Also, it uses pre-defined models which may or may not fully describe the information in the data. Cluster Analysis of Gene Expression Data (CAGED) [29] and Graphical Query Language (GQL) [30] are also useful tools for clustering, but are better suited to longer time series [31]. CAGED provides both an autoregressive approach and a spline linear model based approach and GQL uses hidden Markov models to cluster the data. In the short time series framework, available platforms include the Short Time-series Expression Miner (STEM) [31] and Analysis of Short Time-series using Rank Order preservation (ASTRO) [16]. STEM uses pre-defined profiles to cluster data based on a transformation of the gene profiles to units of change. The user inputs parameters which determine the number of units of change and the number of profiles to consider. Then, clusters are assigned significance levels using a permutation test based method, so not all genes are assigned to significant clusters. ASTRO groups together genes by first constructing a rank matrix for the time series of each gene and then grouping together genes with the same rank profile. Both methods are designed specifically for short time series, and are computational in nature. As such, they transform raw expression data to a sequence of symbols which are then used for clustering. In contrast, PESTS allows the user to select features that are biologically germane to the researcher's interests and sufficiently summarize curve information. It allows flexibility in the number and types of features selected as well as the clustering method. Finally, it provides cluster evaluation metrics which can be used to determine the clustering quality and, by extension, the most appropriate number of clusters to use.

## Conclusion

In this paper, we have introduced PESTS, a software platform for the analysis of time course data. It is designed specifically for the unique methods we have developed for significance analysis, multiple test correction and clustering of short time series data. The central tenet of these methods is the use of biologically relevant features for analysis which summarize gene expression profiles and inherently incorporate the dependence across time. It is fully generalizable to other types of time series analyses. PESTS was designed with a focus on usability and interpretability of analyses for the researcher. As such, it also implements several standard techniques for comparability, as well as visualization functions. These features and functionality make PESTS a valuable resource for a researcher's toolkit.

## Availability and requirements

Project name: PESTS (Processing Expression of Short Time Series)

Project home page: http://www.mailman.columbia.edu/academic-departments/biostatistics/research-service/software-development

Operating system(s): Platform independent

Programming language: Java

Other requirements: Java 6 or higher

License: non-commercial research use license

Any restrictions to use by non-academics: license needed for commercial use

## Abbreviations

ASTRO: Analysis of Short Time-series using Rank Order preservation; CAGED: Cluster Analysis of Gene Expression Dynamics; EDGE: Extraction of Differential Gene Expression; GQL: Graphical Query Language; ORIOGEN: Order Restricted Inference for Ordered Gene Expression data; PESTS: Processing Expression of Short Time Series; STEM: Short Time-series Expression Miner; SAM: Significance Analysis of Microarrays;

## Authors' contributions

AS and MM both contributed to the design of PESTS as well as the implemented algorithms. AS implemented PESTS. AS and MM both participated in the drafting of the manuscript. Both authors read and approved the final manuscript.
